# Loss of stretch-activated channels, PIEZOs, accelerates non-small cell lung cancer progression and cell migration

**DOI:** 10.1042/BSR20181679

**Published:** 2019-03-22

**Authors:** Zhicheng Huang, Zhiqiang Sun, Xueying Zhang, Kai Niu, Ying Wang, Jun Zheng, Hang Li, Ying Liu

**Affiliations:** 1 Department of Radiology, Jilin Province Cancer Hospital, Changchun, Jilin, China; 2 Department of Interventional Radiology, Jilin Province Cancer Hospital, Changchun, Jilin, China; 3 The Third Division of Internal Medicine, Jilin Province Cancer Hospital, Changchun, Jilin, China; 4 Department of Thoracic Oncology, Jilin Province Cancer Hospital, Changchun, Jilin, China

**Keywords:** cell migration, NSCLC, PIEZO, Prognostic roles

## Abstract

PIEZO channels are stretch-activated channels involved in wound sealing and cell proliferation in many cell types. A recent study focussing on lung cancer (LC), using next-generation sequencing analysis, has indicated that PIEZO functions were implicated in LC development. However, the expression and role of PIEZO channels in non-small cell LC (NSCLC) progression require elucidation. In the current study, we investigated the gene expression and alteration frequency in human NSCLC tissue, accessed the prognostic roles of PIEZO channels in NSCLC patients, and further studied the effect of PIEZOs in NSCLC cell proliferation and tumor growth ***in vivo***. The mRNA expression of *PIEZO1* and *2* was clearly decreased in NSCLC tumor tissue compared with that in matched adjacent non-tumor tissue. In human NSCLC tissues, *PIEZO1* gene expression exhibits a highly deep deletion rate, and *PIEZO2* mainly exhibits mutation in gene expression. High mRNA expression of PIEZO channels was found to correlate with better overall survival (OS) for NSCLC patients, especially for patients with lung adenocarcinoma (LUAD), but not for patients with lung squamous cell carcinoma (LUSC). The prognostic role of PIEZO channels was more sensitive in female patients than male patients, and more sensitive in patients at earlier stages than patients at latter stages. Knockdown of *PIEZO1* or *PIEZO2* in NSCLC cells significantly promoted cell migration ***in vitro*** and tumor growth ***in vivo***. These results indicate the critical prognostic values of the PIEZO channels in NSCLC. This information will be beneficial to understand the pathological mechanism of NSCLC and to generate effective therapeutic approaches for NSCLC patients.

## Introduction

Lung cancer (LC) is one of the major causes of deaths worldwide, killing 8.2 million people annually [1]. Like other cancers, it is characterized by the rapid division and uncontrolled growth of cells, in this instance, in lung tissue. Non-small cell LC (NSCLC) represents 85–90% of all LC, and mainly includes lung squamous cell carcinoma (LUSC) and lung adenocarcinoma (LUAD) [1]. Radiation, heavy metals, genotoxic agents, cigarette smoke, and other non-genetic factors are associated with NSCLC [2,3]. In terms of carcinogens, a covalent carcinogen—DNA adduct may result in LC by causing misincorporation leading to genetic mutations [4]. Research focussing on genetic reasons for NSCLC has demonstrated that epidermal growth factor receptor (EGFR) is the most commonly mutated protein that results in LC. Approximately 90% of EGFR mutations in LC are a result of deletion in exon 19 affecting either the conserved sequence LREA (delE746-A750) or Leucine to Arginine at 858 (L858R) [5]*.* However, the molecular mechanism resulting in the pathogenesis of LC is still not fully understood.

*PIEZO* homologs are found in diverse organisms, such as invertebrates, protozoa, and plants [6]. They are non-selective Ca^2+^-permeable cation channels that act as important mediators of various aspects of mechanotransduction [7,8], and with regard to mechanical functions, they are abundant in organs, including the skin, bladder, lungs, and somatosensory dorsal root ganglion (DRG) neurones [9]. Many studies have recently indicated that PIEZO channels are highly expressed in human lung tissue and would be an important factor in lung diseases, especially in LC. However, there is still little understanding of the function of *PIEZO1* and *2* in NSCLC progression [10]. When it is expressed in endothelial cells, *PIEZO1* plays a key role in sensing blood flow-caused shear stress, which is important for blood vessel development [11]. On the other hand, proprioception and touch sensation are mediated by *PIEZO2* and specialized touch receptors that are located in primary sensory neurones and the skin. More importantly, various genetic diseases caused by alteration of channel properties are associated with mutations in human *PIEZO1* and *PIEZO2* genes [12]. *PIEZO1* is able to control epithelial cell crowding and division, determine neural stem cell lineage [13], and regulate blood pressure and exercise performance [14,15]. According to recent research, mutations in the *PIEZO1* gene of humans contribute to anemia (dehydrated stomatocytosis) and generalized lymphatic dysplasia [16,17]. *In vitro* experiments show that knockdown of *PIEZO1* in lung epithelial cells promotes cell migration and reduces cell adherence, suggesting that lack of *PIEZO1* expression may lead to cell migration and metastasis in lung tumors [18]*. PIEZO2* is important in touch sensation and the airway stretch sensation mediated by sensory neurones [19–21]. *PIEZO2* gene mutations are responsible for distal arthrogryposis and other diseases [22,23]. *PIEZO2* knockdown has been shown to promote anchorage-independent growth in premalignant human fibroblasts [24]. Since there are no reports on the prognostic roles of *PIEZO*s in NSCLC patients, we investigated the expression and roles of *PIEZOs* in this patient group.

## Materials and methods

### Analysis of gene alteration frequency in NSCLC

We analyzed the gene alteration frequency of *PIEZO1* and *2* in NSCLC patients from the The Cancer Genome Atlas (TCGA) database using the cBioportal for cancer genomics analysis (http://www.cbioportal.org/) [25].

### Expression of *PIEZO* channels in NSCLC tissues

We queried the expression level of *PIEZO1* and *2* in NSCLC from the Gene Expression Omnibus (GEO), and two original datasets were downloaded (GSE10072 and GSE19804). The differentially expressed mRNA for *PIEZO1* and *2* in NSCLC samples (GSE10072 and GSE19804) and adjacent non-tumor tissues were used for analysis.

### Collection of human LC tissue samples and ethics statement

LC tissues and paired adjacent non-tumor normal lung tissues from NSCLC patients were obtained at Jilin Province Cancer Hospital in 2012, as previously described [26]. All fresh tissues were stored at −80°C until subsequent experiments. The study was approved by the Ethical Committee of Jilin Province Cancer Hospital. An informed consent was obtained from all the participants before enrollment in the study. The entire study was performed based on the Declaration of Helsinki.

### Human LC cell culture

Human LC cell (A549, CCL-185) was obtained from the American Type Culture Collection (ATCC) and cultured as described previously [26]. A549 cells were cultured in Dulbecco’s modified Eagle’s medium (DMEM) supplemented with 10% FBS (Sigma–Aldrich, Inc., St. Louis, MO, U.S.A.) at 37°C in a 5% CO_2_ humidified atmosphere.

### Real-time quantitative PCR analysis of gene expression

Total RNA from human tissue samples and cells was extracted using TRIzol reagent (Invitrogen, Inc; Carlsbad, CA, U.S.A.) and 1 µg of total mRNA was reverse-transcribed into cDNA by using a reverse transcription kit (Bio-Rad, Inc; Hercules, CA, U.S.A.). Real-time quantitative PCR (RT-qPCR) analysis of gene expression used the following primers: *PIEZO1*, forward primer: 5′-GGACTCTCGCTGGTCTACCT-3′; *PIEZO1*, reverse primer: 5′-GGGCACAATATGCAGGCAGA-3′; *PIEZO2*, forward primer: 5′-ATGGCCTCAGAAGTGGTGTG-3′; *PIEZO2*, reverse primer: 5′- ATGTCCTTGCATCGTCGTTTT-3′; glyceraldehyde-3-phosphate dehydrogenase (*GAPDH*), forward primer: 5′-ACAACTTTGGTATCGTGGAAGG-3′; *GAPDH*, reverse primer: 5′-GCCATCACGCCACAGTTTC-3′. RT-qPCR analysis was performed using SYBR Premix ExTaq (Takara). The levels of PCR products were monitored using an Mx3000P QPCR system (Agilent, Santa Clara, CA, U.S.A.). The thermal cycling conditions were as follows: 10 s at 95°C, 40 cycles of 5 s at 95°C, and 30 s at 60°C. The mRNA expression of *PIEZO1* and *PIEZO2* was normalized to the constitutive expression level of *GAPDH* mRNA.

### Antibodies and Western blot analysis

Protease inhibitor cocktail tablets (EDTA-free complete) were from Sigma–Aldrich, Inc. (St. Louis, MO, U.S.A.). Rabbit anti-PIEZO1, anti-PIEZO2, and mouse anti-GAPDH antibodies were purchased from Thermo Fisher Scientific, Inc. (Waltham, MA, U.S.A.). Horseradish peroxidase (HRP)-linked anti-mouse IgG and anti-rabbit IgG antibodies were obtained from Bio-Rad Laboratories, Inc. (Hercules, CA, U.S.A.). Immunoblot analysis was performed as described previously [26]. Briefly, cell lysates were prepared in lysis buffer containing EDTA-free complete protease inhibitors, followed by centrifugation at 10000×***g*** for 10 min, and boiled with Laemmli sample buffer for 5 min. Cell lysates (20 µg protein) were separated on 10% or 4–20% SDS/PAGE, then transferred to polyvinylidene difluoride (PVDF) membranes, and blocked with TBST containing 5% BSA prior to incubation with primary antibodies (1:1000 dilution) overnight, and secondary antibodies (1:2000 dilution) for 2 h at room temperature. Blots were developed using the ECL chemiluminescence kit, and the integrated density of pixels in each membrane was quantitated using Image Quant 5.2 software (Molecular Dynamics, Sunnyvale, CA, U.S.A.).

### Prognostic role of PIEZO expression in NSCLC

We used a public database to study the relevance and significance of the *PIEZO* expression level to overall survival (OS) in NSCLC patients. The data regarding NSCLC patients used for the Kaplan–Meier (KM) plotter analysis were pooled from TCGA, (http://cancergenome.nih.gov), GEO (http://www.ncbi.nlm.nih.gov/geo/), European genome-phenome archive (EGA) (https://ega.crg.eu/), and PubMed (http://www.pubmed.com) [27]. The database included the mRNA expression of two PIEZO channels and survival information (20 years) from 1432 NSCLC patients.

To obtain KM plots for patient OS rates, we used the KM plotter database (http://kmplot.com/analysis/index.php?p=service&cancer=lung). Based on the expression of genes, the patients were divided into two groups (high expression: mRNA expression higher than the median separates; low expression: mRNA expression lower than the median separates). Hazard ratio (HR), 95% confidence intervals, and log rank *P*, were also calculated from the database, and are included in the figures and tables of this manuscript. *P*<0.05 was used to indicate a statistically significant difference [28].

### shRNA transfection

Vehicle control shRNA (Veh shRNA, SHC016), human *PIEZO1* sh-RNA (sh-PIEZO1, TRCN0000121969), and *PIEZO2* sh-RNA (sh-PIEZO2, TRCN0000123253) were purchased from Sigma–Aldrich, Inc. (St. Louis, MI, U.S.A.). A549 cells cultured on six-well plates (50–60% confluence) were transiently transfected with Veh sh-RNA or sh-PIEZO1 or sh-PIEZO2 (3 µg/well) using a plasmid transfection kit (Qiagen, Inc., Gaithersburg, MD, U.S.A.) according to the manufacturer’s instructions. Forty-eight hours post-transfection, cells were selected and cultured in growth medium containing puromycin (1 µg/ml). Two weeks post-transfection, the expression of *PIEZO1* and *2* in cells was analyzed using RT-qPCR.

### Scratch assay

A549 cells, with stable transfection of shRNA (Veh shRNA, sh-PIEZO1, or sh-PIEZO2), were grown to confluence in complete DMEM containing 1 µg/ml puromycin. At time 0 h, a 2-mm scrape wound was created with a pipette tip. Dead and floating cells were washed out from the complete medium. The remaining cells were continuously cultured in complete medium containing Mitomycin C (1 μg/ml) [29]. Cell migration was recorded at 0 and 24 h from at least three independent experiments. Wound healing was quantitated using Image Quant 5.2 software (Molecular Dynamics, Sunnyvale, CA, U.S.A.) and normalized with the wound healing in the cells transfected with Veh shRNA. Briefly, % of wound healing = (wound area at 0 h – wound area at 24 h)/wound area at 0 h × 100% [30].

### Mice and *in vivo* tumor growth

Nude mice (female, 6 weeks old) were obtained from the Animal Center of the Chinese Academy of Science (Shanghai, China), and maintained in the nude mice care center of the Jilin Cancer Hospital. A549 cells with stable transfection of Veh shRNA, sh-PIEZO1, and sh-PIEZO2 were subcutaneously injected into nude mice (1 × 10^7^ cell/ml, 50 µl). After injection, the tumor nodules were measured every week with a caliper. The tumor volume was calculated using: tumor volume (mm^3^) = π/6 × a × b^2^, where a is the longest diameter and b is the shortest diameter. During the 8-week follow-up period, the tumor size of nude mice was recorded.

### Statistical analysis

All data are expressed as means ± S.E.M. from at least three independent experiments. Results were subjected to statistical analysis using one-way ANOVA or two-tailed Student’s *t* test as described before [26]. Values of *P*<0.05 were considered significant.

## Results

### Decreased expression of *PIEZO* channels in NSCLC

**Figure 1:**
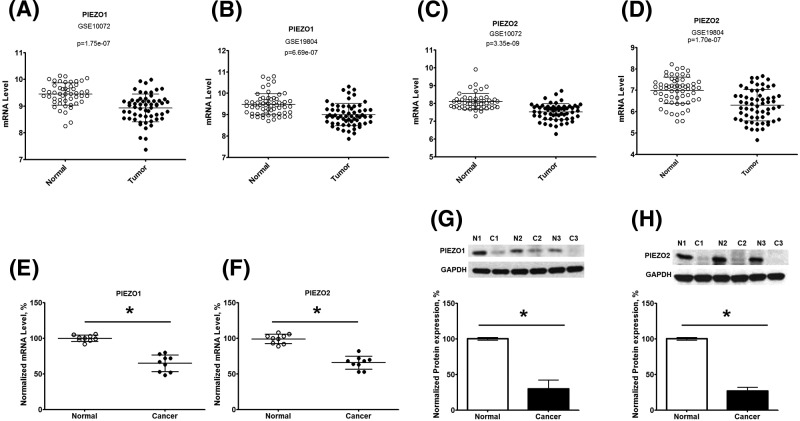
Expression of *PIEZO1* and *2* in normal and tumor tissues from human NSCLC patients mRNA and protein expression of PIEZO1 and 2 in lung tumors was compared with the expression in adjacent normal tissues. (**A**,**B**) Expression levels of *PIEZO1* in human lc tissues compared with normal tissues in GSE10072 (A) and GSE19804 (B). (**C**,**D**) Expression levels of *PIEZO2* in human LC tissues compared with normal tissues in GSE10072 (C) and GSE19804 (D). (**E**,**F**) mRNA expression of *PIEZO1* (E) and *PIEZO2* (F) in cancer tissue and adjacent normal tissues from human NSCLC patients were collected from Jilin Hospital. The mRNA expression was analyzed by RT-qPCR, and normalized to the expression of GAPDH. (**G**,**H**) Protein expression of *PIEZO1* (G) and *PIEZO2* (H) in cancer tissue and adjacent normal tissues. Upper panel indicates the representative images of Western blot from cancer tissue (C1–C3) and adjacent normal tissues (N1–N3), and the lower panel shows the quantitation of protein expression (*n*=5). The normalized expression of *PIEZO1* and *PIEZO2* is displayed as mean ± S.D. *P<0.05.

**Figure 2:**
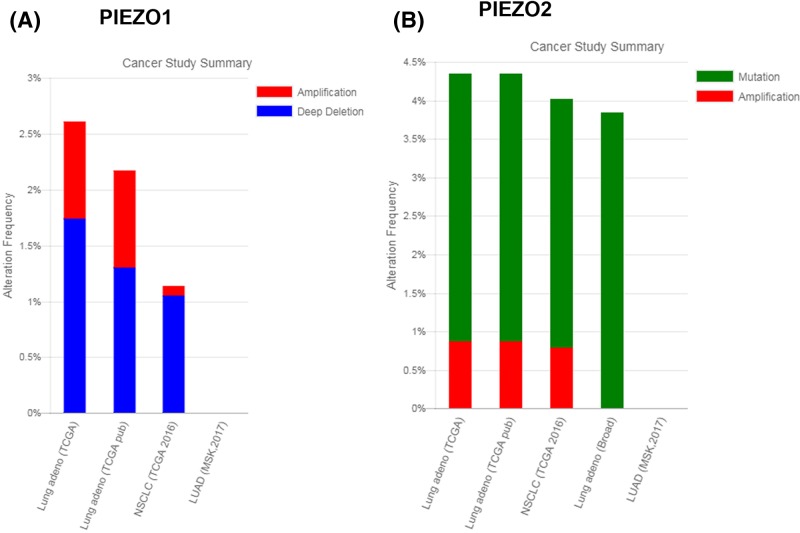
Alteration frequency of *PIEZO1* and *2* in NSCLC The alteration of *PIEZO1* and *2* were visualized using the cBioPortal for Cancer Genomics database. Mutation, deletion, and amplification are shown in different colors. (**A**) Alteration frequency of *PIEZO1* in NSCLC. (**B**) Alteration frequency of *PIEZO2* in NSCLC.

First, we analyzed the mRNA expression of *PIEZO1* and *2* in NSCLC tissues and normal tissues from microarray. Raw microarray data were retrieved using the search terms ‘GSE#10072’ and ‘GSE19804’ in the GEO dataset. The analysis indicated that mRNA expression of *PIEZO1* and *2* in NSCLC tissues was significantly lower than that in non-tumor tissues (*P*<0.0001; Figure 1A–D). Next, we used RT-qPCR to analyze the mRNA and protein expression of *PIEZO1* and *PIEZO2* in LC and adjacent control tissues from NSCLC patients, which were collected from our hospital in 2012. The mRNA expression of *PIEZO1* and *2* in human tissues was analyzed by RT-qPCR and normalized to the expression of GAPDH. As shown in Figure 1E–H, the mRNA (**E**,**F**) and protein (**G**,**H**) expression of *PIEZO1* (Figure 1E,G) and *PIEZO2* (Figure 1F,H) in cancer tissue from NSCLC patients were significantly lower than that in the adjacent non-cancer tissues. Next, we analyzed the gene alteration frequency of *PIEZO1* and *2* in NSCLC tissues. We found that there was a high deep deletion rate of *PIEZO1* gene in NSCLC (Figure 2A), and a high gene mutation rate of the *PIEZO2* gene in NSCLC (Figure 2B). These results indicate that *PIEZO1* and *2* are decreased in NSCLC tissues in comparison with matched adjacent non-tumor tissue, which may be due to gene alteration of PIEZO channels during NSCLC progression.

### Association between the expression *of PIEZO* channels and clinicopathological characteristics of NSCLC patients

**Figure 3:**
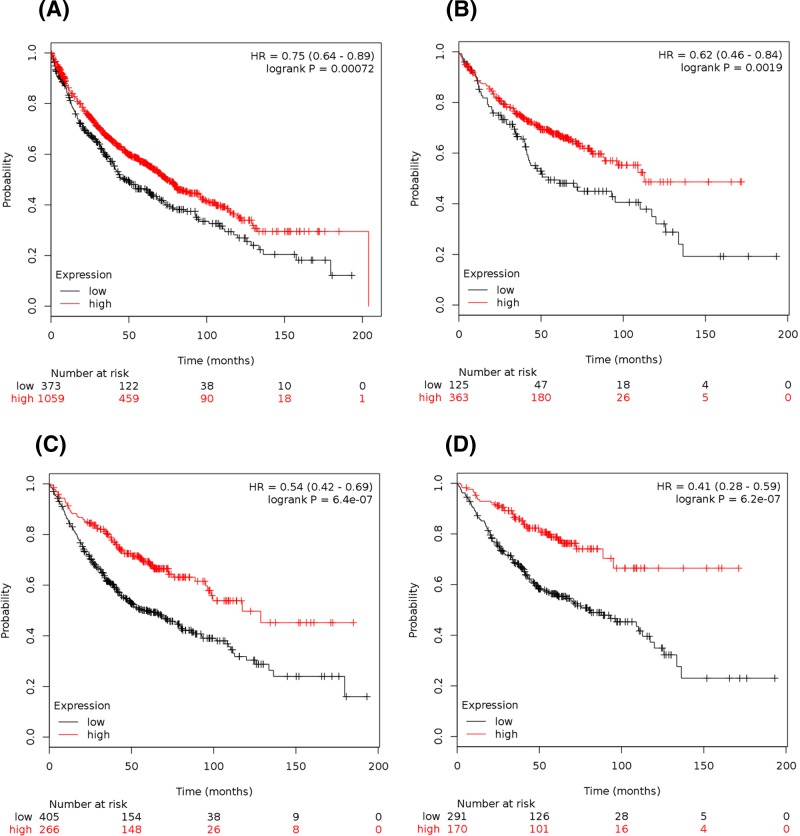
Prognostic value of the expression *of PIEZOs* in NSCLC (**A**,**B**) Prognostic value of *PIEZO1* expression in NSCLC patients (A, *n*=1432) and LUAD patients (B, *n*=488). (**C**,**D**) Prognostic value of *PIEZO2* expression in NSCLC patients (C, *n*=671) and LUAD patients (D, *n*=461).

**Figure 4:**
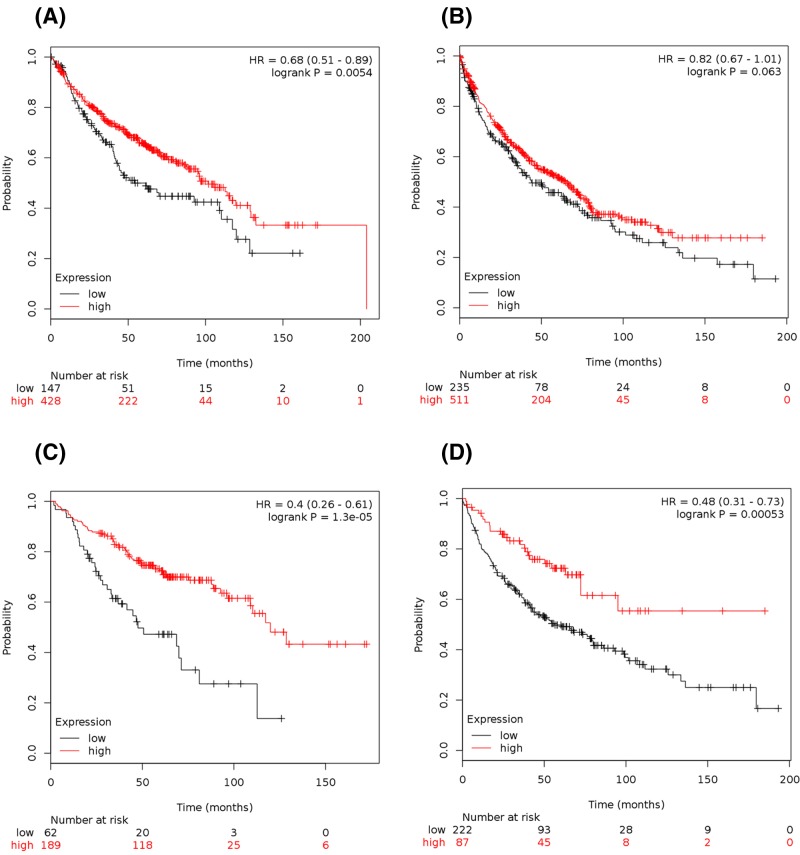
Prognostic value of the mRNA expression of *PIEZOs* in female and male patients (**A**,**B**) Prognostic value of *PIEZO1* mRNA expression in female patients (A, *n*=575) and male patients (B, *n*=746). (**C**,**D**) Prognostic value of *PIEZO2* mRNA expression in female patients (C, *n*=251) and male patients (D, *n*=309).

We used the KM plotter to determine the prognostic value of *PIEZOs* in the database. The Affymetrix IDs is valid: 202771_at (*PIEZO1*) and 222908_at (*PIEZO2*). Survival curves were drafted for *PIEZO1* in all NSCLC patients (*n*=1432) (Figure 3A), LUAD patients (*n*=488) (Figure 3B), and LUSC patients (*n*=421) (data not shown). The high expression of *PIEZO1* mRNA was correlated to better OS for all NSCLC patients, HR 0.79 (0.64–0.89), *P*=0.00072 (Figure 3A). In particular, the high expression of *PIEZO1* mRNA was strongly correlated to better OS in LUAD patients, HR 0.62 (0.46–0.84), *P*=0.0019 (Figure 3B), but not in LUSC patients, HR 0.86 (0.65–1.12), *P*=0.26 (data not shown). The high expression of *PIEZO1* mRNA was also strongly correlated to better OS in female patients, HR 0.68 (0.51–0.89), *P*=0.0054 (Figure 4A), but not in male patients, HR 0.82 (0.67–1.01), *P*=0.063 (Figure 4B).

For *PIEZO2*, the survival curves were drafted in all NSCLC patients (*n*=671) (Figure 3C), in LUAD patients (*n*=461) (Figure 3D), and in LUSC patients (*n*=168) (data not shown). The high expression of *PIEZO2* mRNA was correlated to better OS for all NSCLC patients, HR 0.54 (0.42–0.69), *P*=6.4 x 10^-7^ (Figure 3C). Interestingly, the high expression of *PIEZO2* mRNA was strongly correlated to better OS in LUAD patients, HR 0.41 (0.28–0.59), *P*=6.2e-7 (Figure 3D), but not in LUSC patients, HR 1.39 (0.88–2.2), *P*=0.15 (data not shown). The high expression of *PIEZO2* mRNA was strongly correlated to better OS in female patients, HR 0.4 (0.26–0.61), *P*=1.3 x 10^-5^ (Figure 4C), and in male patients, HR 0.48 (0.31–0.73), *P*=0.00053 (Figure 4D).

To further assess the association of *PIEZOs* with other clinicopathological profiles, we determined the correlation with the smoking status of the patients, different clinical stages, and surgical treatments (Table 1). As shown in Table 1, the high expression of *PIEZO1* and *2* was strongly correlated with better OS in non-smoking patients, but not in patients who smoked. From Table 1, the low mRNA expression of *PIEZO1* was associated with worse OS of patients with grades I and II, but not with grade III. The low mRNA expression of *PIEZO2* was associated with worse OS of patients with grades I, II, and III. From Table 1, the low mRNA expression of *PIEZO2*, but not *PIEZO1*, was associated with worse OS in patients with negative surgical margins.

**Table 1:** Correlation of gene expression with NSCLC patients in different clinical variables

Clinical variables		Genes	Cases	HR	95% CI	*P*-value
Smoking status	Never smoked	PIEZO1	178	0.5	0.25–0.971	0.037
	Smoked	PIEZO1	669	1.14	0.9–1.46	0.28
	Never smoked	PIEZO2	117	0.37	0.11–1.32	0.11
	Smoked	PIEZO2	164	0.48	0.24–0.95	0.032
Clinical stage	I	PIEZO1	440	0.54	0.38–0.77	0.00046
	II	PIEZO1	185	0.51	0.31–0.84	0.0067
	III	PIEZO1	67	1.42	0.72–2.811	0.31
	I	PIEZO2	322	0.38	0.25–0.57	1.1 x 10^-6^
	II	PIEZO2	107	0.56	0.32–1	0.045
	III	PIEZO2	44	2.52	1.1–5.78	0.024
Negative surgical margins	PIEZO1	726	1.14	0.89–1.45	0.29	
	PIEZO2	204	0.28	0.13–0.62	0.00079	

### Knockdown of *PIEZO* genes promotes cell migration and tumor growth

**Figure 5:**
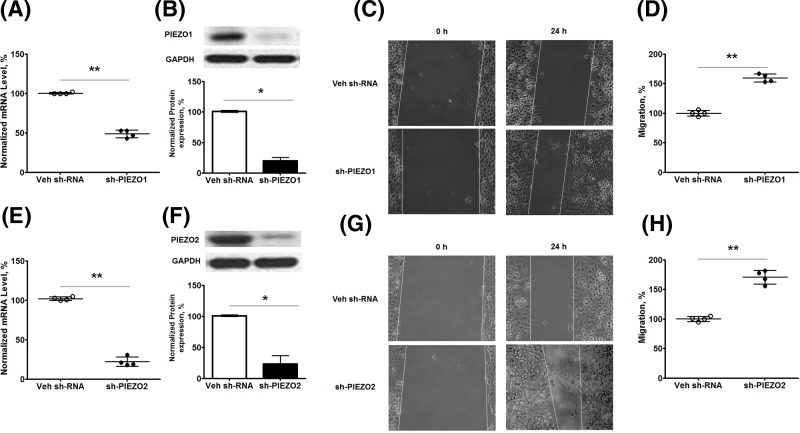
Knockdown of *PIEZO* gene expression promotes cell migration in A549 cells A549 cells were transfected with Veh shRNA and shRNA target *PIEZO1* or *PIEZO2*, and the stable cell line was selected and maintained in culture medium with puromycin as described in the ‘Materials and methods’ section. The mRNA expression of *PIEZO1* and *2* in these cells was analyzed using RT-qPCR, and the protein expression of GAPDH, *PIEZO1*, and *2* in these cells was analyzed using Western blot. Cell migration in A549 cells with and without knockdown of *PIEZO* genes was analyzed by scratch assay as described in the ‘Materials and methods’ section. (**A**,**E**) mRNA expression of *PIEZO1* (A) and *PIEZO2* (E) in A549 cells. (**B**,**F**) Protein expression *PIEZO1* (B) and *PIEZO2* (F) in A549 cells; upper panel indicates the representative images of Western blot, and the lower panel shows the quantitation of protein expression. (**C**,**D**) Cell migration in A549 cells with stable knockdown of *PIEZO1*. Representative images (C) and quantitation of migration (D) in A549 cells with stable knockdown of *PIEZO1*. (**G**,**H**) Cell migration in A549 cells with stable knockdown of *PIEZO2*. Representative images (G) and quantitation of migration (H) in A549 cells with stable knockdown of *PIEZO2*. Data are displayed as mean ± S.D. of gene expression in different cells. *, *P*<0.05; **, *P*<0.01, cells with stable knockdown of *PIEZO1* or *2* compared with cells with stable transfection of Veh shRNA.;

**Figure 6:**
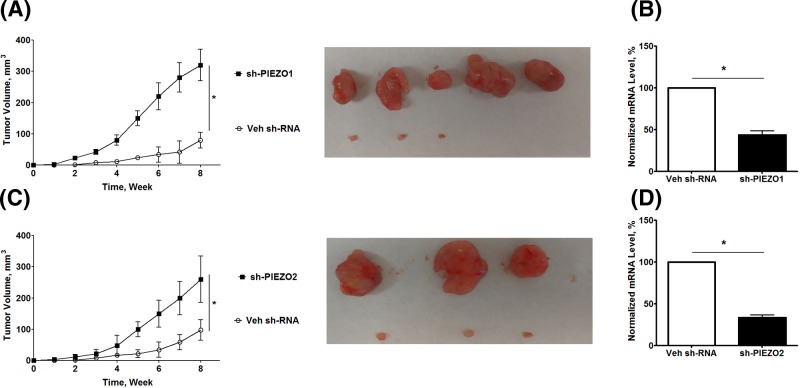
Knockdown of the expression of *PIEZOs* promotes tumor growth *in vivo* Nude mice were subcutaneously inoculated with A549 cells stably transfected with *sh-PIEZO1* (*n*=8), *sh-PIEZO2* (*n*=8), or *Veh shRNA* (*n*=8). The subcutaneous tumor size was recorded every week. Data are presented as mean ± S.D. of the measurement of each group. The subcutaneous tumors were isolated and measured. After inoculation, the tumor volumes were measured every week. *P<0.05. (**A**) Tumor growth in nude mice inoculated with A549 cells with stable knockdown of *PIEZO1*. Right panel shows the *xenografts* from mice with subcutaneous inoculation of A549 cells with or without knockdown of PIEZO1. *sh-PIEZO1* (*n*=5) and *Veh shRNA* (*n*=3). (**B**) qPCR quantitation of mRNA expression of human PIEZO1 from the *xenografts* with or without stable knockdown of PIEZO1. (**C**) Tumor growth in nude mice inoculated with A549 cells with stable knockdown of *PIEZO2*. Right panel shows the *xenografts* from mice with subcutaneous inoculation of A549 cells with or without knockdown of PIEZO2. *sh-PIEZO1* (*n*=3) and *Veh shRNA* (*n*=3). (**D**) qPCR quantitation of mRNA expression of human PIEZO2 from the *xenografts* with or with stable knockdown of PIEZO2. *, *P*<0.05, cells with stable knockdown of *PIEZO1* or *2* compared with cells with stable transfection of Veh shRNA.

To investigate the potential mechanism of PIEZO in the development of NSCLC, we further studied the effects of PIEZO channels in NSCLC cell migration. As shown in Figure 5A,B,E, F, stable transfection of *sh-PIEZO1* (Figure 5A,B) or *sh-PIEZO2* (Figure 5E,F) significantly reduced the mRNA and protein expression of the target gene in A549 cells. In comparison with cells with transfection of Veh *sh-RNA*, cells with stable knockdown of *PIEZO1* (Figure 5C,D) or *PIEZO2* (Figure 5G,H) promoted cell migration in A549 cells. To check the role of PIEZOs in tumor growth, we inoculated *A549* cells with stable transfection of Veh sh-RNA, sh-PIEZO1, or sh-PIEZO2 into nude mice. As shown in Figure 6A, the tumors derived after inoculation with sh-PIEZO1-transfected A549 cells grew faster and larger than the mice inoculated with Veh sh-RNA transfected A549 cells *in vivo* (Figure 6A). The qPCR data indicate that the *xenografts* from mice inoculated with A549 cells with stable knockdown of PIEZO1 showed a greatly decreased expression of human PIEZO1 in comparison with those xenografts grown from A549 cells with stable transfection of Veh sh-RNA (Figure 6B). Similarly, inoculation of A549 cells with stable transfection of sh-PIEZO2 also promoted tumor growth in nude mice (Figure 6C), and these xenografts showed less expression of human PIEZO2 (Figure 6D) in comparison with mice inoculated with control cells. These data indicate that the lower expression of PIEZOs resulted in worse OS in NSCLC patients, at least partly, through promoting cancer cell migration and tumor growth.

## Discussion

PIEZO channels, including *PIEZO1* and *PIEZO2*, represent a new class of mechanosensitive channels, which respond to mechanical forces and allow Ca^2+^ to enter or exit the cell. *PIEZO* channels are large transmembrane proteins with more than 2500 amino acids and 24–36 putative transmembrane segments [31]. PIEZO channels are widely expressed in human tissues. Recent investigations using knockout mice have indicated the physiological importance of *PIEZO* channels in various mechanotransduction processes, such as touch, proprioception, hearing, and blood pressure regulation in mammals. In particular, in endothelial cells, *PIEZO1* plays an essential role in shear stress-sensing caused by blood flow, which is important for proper blood vessel development [32]. In epithelial cells, *PIEZO1* regulates cell crowding and division [14]. *PIEZO1* also regulates blood pressure and exercise performance [15], and determines neural stem cell lineage [13]. *PIEZO2* is important in touch sensation [21,33], mainly expressed in primary sensory neurones [34]. In the airway, *PIEZO2* localized in sensory neurones is critical for the airway stretch sensation [19]. Furthermore, mutations in human *PIEZO1* and *PIEZO2* genes have been linked to various genetic diseases due to alterations in channel properties [22].

In many organs, mechanical cues are converted into biological signals through the detection of mechanotransduction via mechanosensitive channels, such as *PIEZO1* and *2*. Mechanosensitive channels are essential to mechanotransduction and are frequently located on the cell surface. Accumulated research indicates that the regulation of mechanosensitive adaptors is associated with the pathogenesis of various diseases in many organs. In the midgut of adult *Drosophila*, mechanical stress regulates stem cell differentiation through the stretch-activated ion channel PIEZO, which increases cytosolic Ca^2+^ in response to a direct mechanical stimulus [35]. In fibrotic tissues, mechanical signaling acts through the discoidin domain receptor 1 to regulate the interactions of cells with the extracellular matrix (ECM) [36]. The composition and physical property changes of ECM are associated with tumor progression in many organs including the lung [37]. Recently, a number of physiological functions of PIEZO channels have been identified through various biological researches. PIEZOs were proved to sense the stiffness of the surrounding substrate and respond to light touch in a number of different cells [38]. Stiffness plays a key role in regulating the matricellular protein CCN1/CYR61 in endothelial cells during tumor metastasis, suggesting that target stiffness‐induced changes is a potential mechanism to impair tumor metastasis [39]. The matrix stiffness of cancer tissue also affects the phenotypes and properties of many types of cancer cells. In LC cells, a stiff substrate was shown to enhance programmed death-ligand 1 (PD-L1) expression and regulate tumor growth [40]. Human *PIEZO1* gene mutations resulted in anemia and generalized lymphatic dysplasia [16,17], and *PIEZO2* gene mutations were proved to cause distal arthrogryposis and other diseases [22,23]. A recent study has shown that *PIEZO1* regulates epithelial restitution and cell mobility in gastric cancer cells through interaction with trefoil factor family 1 (TFF1), a member of the TFF-domain peptide family [41]; and knockdown of *PIEZO1* expression reduces cell migration in gastric cancer cell lines [42]. In small cell LC (SCLC) cell lines, the expression of PIEZO1 was lower in the SCLC cell lines in comparison with normal control cells [18]. Knockdown of the expression of PIEZO1 in SCLC cells, promoted cell migration and colony numbers in soft agar [18]. Similarly, in our study, we also found that the expression of PIEZO1 and PIEZO2, in the lung tissue from NSCLC patients, and knockdown of the expression of PIEZO1 or PIEZO2 increased cell migration in A549 cells (NSCLC cell line) in the 2D culture system.

*PIEZO1* and *2* are highly expressed in lung tissue. Knockdown of *PIEZO1* in lung epithelial cells attenuated cell adherence and stimulated cell migration [18]. In particular, how lung mechanotransduction regulates lung tumor progression remains elusive. We investigated the expression and prognostic roles of PIEZO channels in NSCLC patients. Our data indicate that the expression of PIEZOs decreased in lung tumor tissues, which may result in the impaired function of *PIEZO* channels in LC patients [6]. Similar to LC, the expression of *PIEZO1* is also down-regulated in radiation-induced thyroid tumors [43]. The potential molecular mechanisms leading to the decreased expression of PIEZOs were different. For *PIEZO1*, gene expression shows high frequency of deep deletion in human LC tissue. However, *PIEZO2* expression shows a high mutation ratio in cancer tissue from NSCLC patients. The predicted roles of PIEZOs in NSCLC patients, both *PIEZO1* and *2*, are those involved in the suppression of tumor progression. The higher expression of *PIEZO1* and *PIEZO2* was correlated to better OS for all NSCLC patients. Indeed, we provide compelling evidence that *PIEZO1* and *2* have critical prognostic values in NSCLC. Meanwhile, Yoda1, an activator for activation or sensitization of membrane tension, and non-specific blockers of PIEZOs (such as gadolinium, ruthenium red, and streptomycin), have been reported to regulate PIEZO functions [44], which might be useful in understanding the heterogeneity and complexity of the molecular biological role of PIEZOs in the pathogenesis of NSCLC and other diseases. Our current study indicated that blocking the function of PIEZOs or knockout of the expression of PIEZOs promotes tumor formation, which may suggest that activators targetting PIEZOs would be a potential candidate for the treatment of NSCLC.

In summary, our investigation demonstrates that the expression of PIEZOs decreased in NSCLC tissues, which may be due to the alteration of gene expression. High mRNA expression of PIEZO channels was found to correlate with better OS for all NSCLC patients, especially for LUAD patients, which may be due to the regulation of cell migration and tumor growth. Our further investigation of the prognostic roles of PIEZO channels in different clinicopathological features indicated that there are critical prognostic values of *PIEZO1* and *2* channels in NSCLC. This information suggests that *PIEZO1* and *2* might be a potential drug target for NSCLC patients, and the expression of these genes would be useful to develop novel tools to effectively predict the prognosis of NSCLC.

## Abbreviations

DMEM: Dulbecco’s modified Eagle’s medium
ECM: extracellular matrix
EGFR: epidermal growth factor receptor
GAPDH: glyceraldehyde-3-phosphate dehydrogenase
GEO: Gene Expression Omnibus
HR: hazard ratio
KM plotter: Kaplan–Meier plotter
LC: lung cancer
LUAD: lung adenocarcinoma
LUSC: lung squamous cell carcinoma
NSCLC: non-small cell LC
OS: overall survival
RT-qPCR: real-time quantitative PCR
SCLC: small cell LC
TBST: Tris-buffered saline with 0.1% Tween 20
TCGA: The Cancer Genome Atlas
